# Supplementary material for: High Energy Density of Ball-Milled Fluorinated Carbon Nanofibers as Cathode in Primary Lithium Batteries
Source: Nanomaterials (Basel). 2024 Feb 22;14(5):404. doi: 10.3390/nano14050404 (PMC10933857; doi:10.3390/nano14050404)
Supplement: Supplementary file 1 [file nanomaterials-14-00404-s001.zip › nanomaterials-2797996-supplementary.pdf]

nanomaterials

Type of the Paper (Article)

Supplementary Information

High energy density of ball-milled fluorinated carbon nanofibers as cathode in primary lithium batteries

Marie Colin[1], Elodie Petit[1], Katia Guérin[1], Marc Dubois[1,*]

[1] Université Clermont Auvergne, SIGMA Clermont, CNRS, Institut de Chimie de Clermont-Ferrand (UMR 6296), BP 10448, F-63000, Clermont-Ferrand, France
* Correspondence: marc.dubois@uca.fr

Scanning electron microscopy images of F-Gr compounds before and after milling are shown in Figure SI1. F-Gr have an average particle size of around 4 µm. After Grinding, their size was reduced and the pieces aggregated.

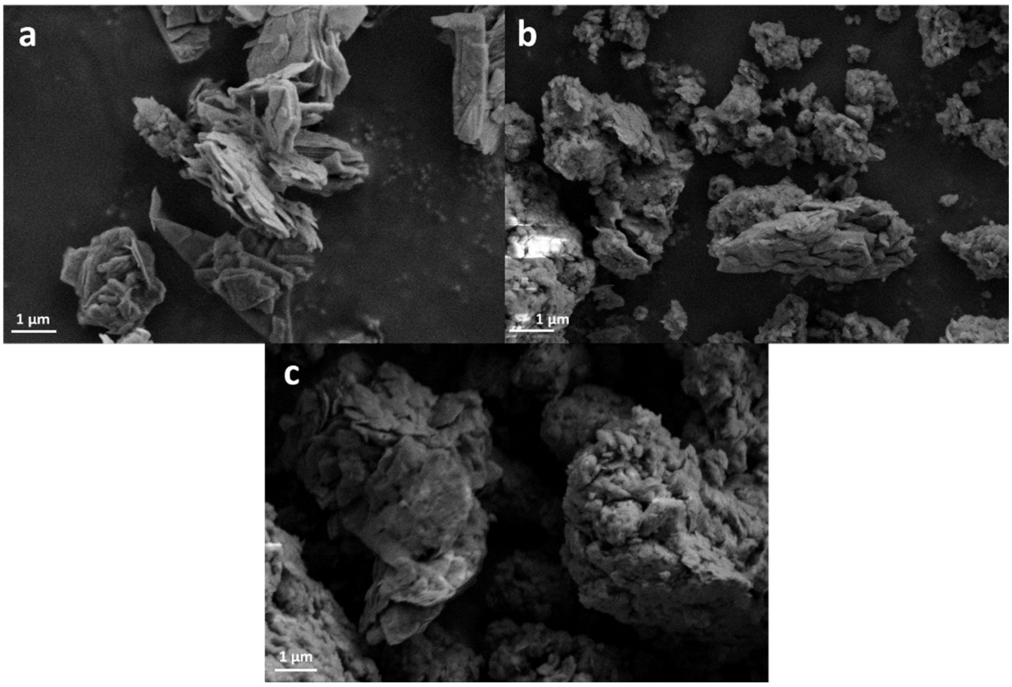

Figure S1: SEM images of fluorinated graphites before Grinding (a), F-Gr after grinding in air (b) and in argon (c).

X-ray diffractoGrams of fluorinated graphite samples before and after milling are shown in Figure SI2. Each material shows a first diffraction peak centered at an angle around 14° corresponding to the diffraction plane (001) characterizing the distance between the fluorocarbon planes. This distance is 0.61 nm for F-Gr, 0.62 nm for F-Gr-air and F-GR-Ar, typical of a (CF)n fluorinated phase. It remains unchanged in the case of GR Grinding under

Citation: To be added by editorial staff during production.

Academic Editor: Firstname Lastname

Received: date
Revised: date
Accepted: date
Published: date

Copyright: © 2023 by the authors. Submitted for possible open access publication under the terms and conditions of the Creative Commons Attribution (CC BY) license (https://creativecommons.org/licenses/by/4.0/).

the conditions employed. The second diffraction peak at 21.7°, present for all samples, is substrate-related. The (100) plane also diffracts at 40.8° for all samples. Graphite samples ground under argon and air show a diffraction peak at 73° (110) [46].

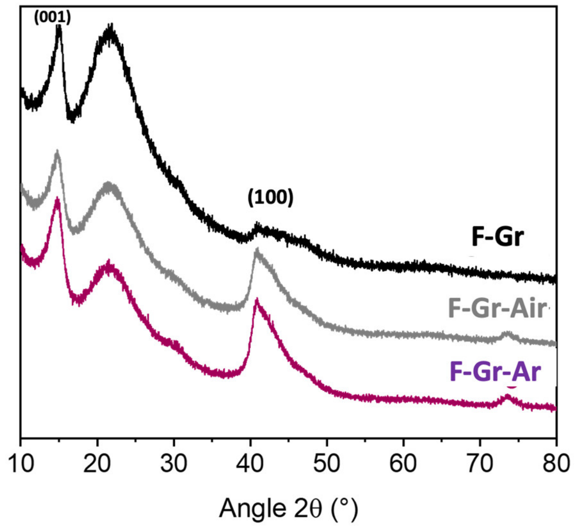

Figure S2. X-ray diffractograms of fluorinated graphites before and after Grinding

The spectra of F-Gr before and after Grinding are shown in Figure 12. The spectra of all three compounds are characterized by a vibrational band for covalent C-F bonds at 1190 cm^{-1} and a slight shoulder at 1076 cm^{-1} for weaker C---F bonds. The covalence of C-F bonds does not appear to be altered by grinding. CF_2 Groups (1350 cm^{-1}) are present in all three compounds. A band at 1876 cm^{-1} attributed to C=O bonds with a fluorinated environment (CO-F) is present for F-Gr-air; oxygen is supplied by the milling atmosphere.

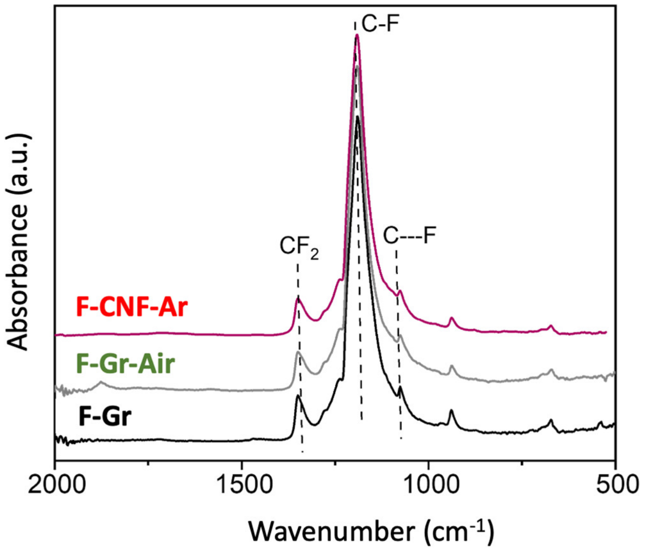

Figure S3. Infrared spectra of fluorinated GR compounds before and after Grinding

The ^{19}F solid state NMR spectra of GR compounds before and after milling are shown in Figure SI4. The spectra of all three fluorinated graphites show a fine peak centered at -187 ppm attributed to C-F bonds, with CF_3 groups visible at -78 ppm and CF_2 groups at -116

ppm. Attribution of the band at -125 ppm to C-F bonds with an oxygenated environment, as in the case of fluorinated Graphene oxide [SI1], was not retained in view of the infrared spectra which did not demonstrate their presence. The F-Gr-air compound contains oxygen in the form of CO-F bonds, as demonstrated by infrared analysis (Figure SI3). The chemical shifts of the various contributions and the intensity of the peaks remain the same before and after Grinding.

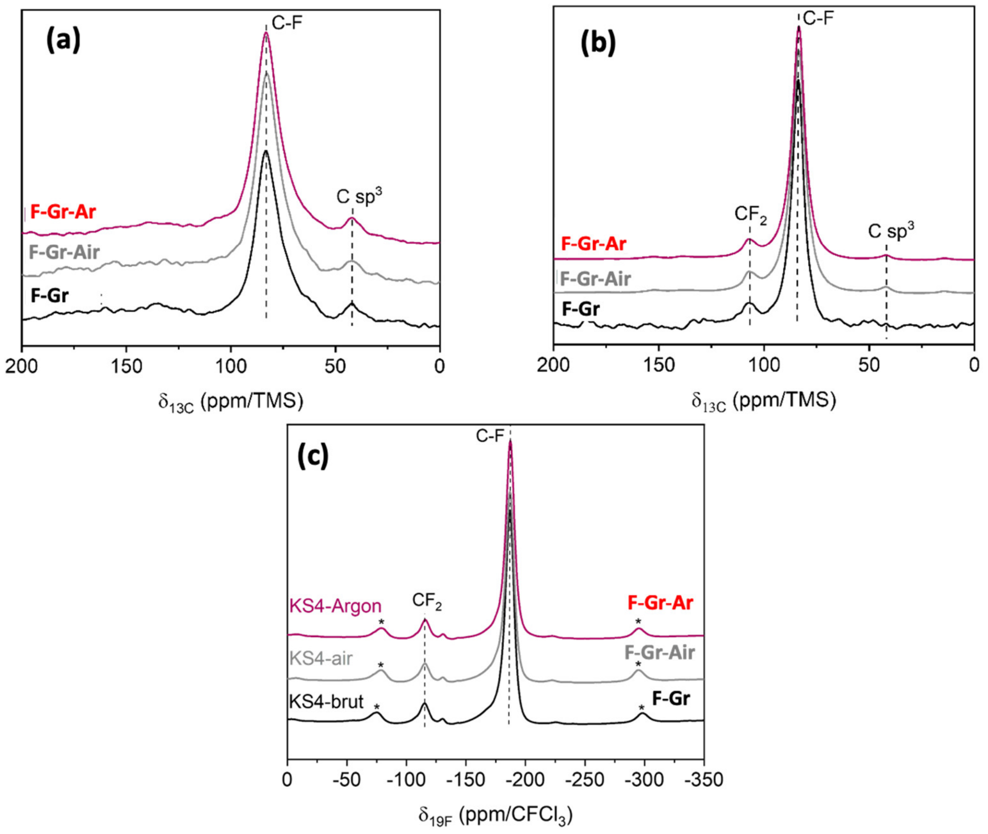

Figure S4. ^{13}C (a), CP $^{19}F \rightarrow ^{13}C$ (b) and ^{19}F MAS NMR spectra of fluorinated graphites before and after Grinding.

In ^{13}C NMR (Figure SI4a), C-F bonds are highlighted by a peak at 83 ppm. The C-F bonds remain covalent even after milling. The F/C fluorination rate is 0.80 ± 0.02 for F-Gr, 0.78 ± 0.02 for F-Gr-air and 0.83 ± 0.02 for F-Gr-Ar. Grinding in either air or Argon did not significantly alter the fluorine content of the material. Sp^3 C are evidenced by the peak at 42 ppm for all three samples.

With a $^{19}F \rightarrow ^{13}C$ cross-polarization sequence amplifying signals from fluorine-bonded carbons (Figure 14 b), the spectra show no difference in C-F resonance, which is centered at 83 ppm for all three samples. CF_2 is present at 110 ppm in small proportions

Galvanostatic discharge curves at 10 mA/g up to 1.5 V for compounds before and after Grinding are shown in Figure SI5.

F-Gr and F-Gr-Ar show galvanostatic discharge curves with a flat profile, typical of CFx but not regular. The delivered capacities are 848 mAh/g for F-Gr($C_{Theoretical}$ = 788 mAh/g considering the fluorination rate established by ^{13}C NMR) and 818 mAh/g for F-Gr-Ar ($C_{Theoretical}$ = 801 mAh/g). As the capacities obtained are close to the expected theoretical capacities (within the experimental error, i.e. ± 10%), all available C-F bonds were involved

in the electrochemical reduction. The reduction potentials are 2.30 V and 2.34 V for F-Gr and F-Gr-Ar, respectively. A slight increase in potential was observed after milling under argon.

F-Gr-Ar was tested at different current densities, as F-Gr, to study the effect of milling on the electrochemical performances. Galvanostatic discharge curves at different cruurent rate are shown in Figure SI5c. The current densities applied according to the discharge regimes and the experimental electrochemical data are summarized in Table 3 and Table 4.Discharge curves for GR-Gross at higher current densities show irregular profiles, with a reduction potential that decreases drastically from 0.05C onwards. Capacities delivered remain correct, but the discharge plateau is irregular, which is undesirable for a real device. The F-Gr-Ar has difficulty delivering significant capacity at low currents (0.05C and 0.2C), but delivers 493 mA/h at 1C. The average reduction potential at 1C is higher after milling under Argon: it is 1.90 V versus 1.68 V for F-Gr, but the usual flat profile of the discharge curves is no longer present.

The power tests using the discharge curves of F-Gr and F-Gr-Ar are difficult to obtain repeatedly. The loss of capacity with increasing discharge rates is significant: only 26% of the theoretical capacity is delivered for F-Gr and 61% for F-Gr-Ar at 1C.

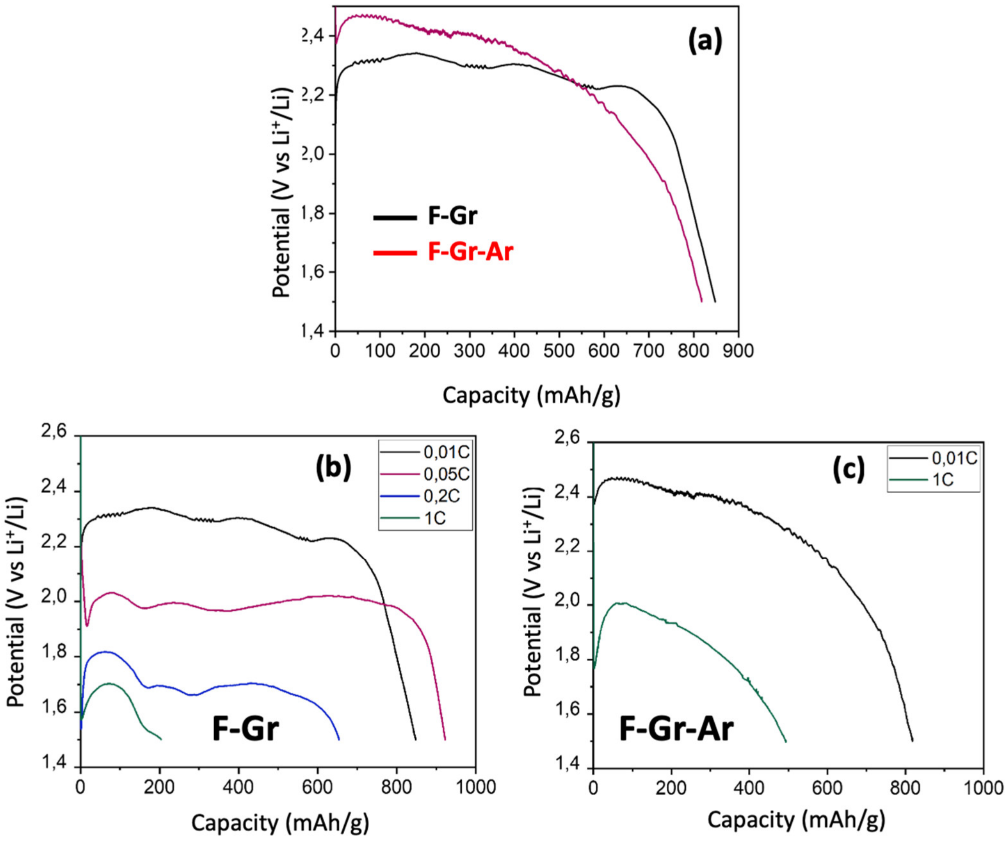

Figure S5. Galvanostatic discharge curves for F-Gr (a) and F-Gr-Ar (b) at different current densities with 1M $LiPF_6$ in EC/PC/3DMC electrolyte

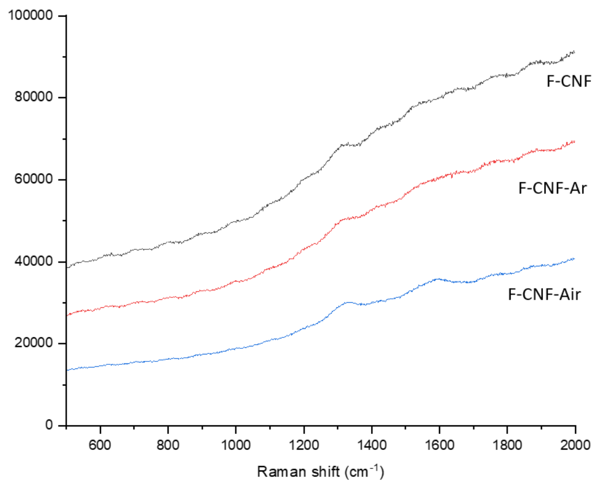

Figure S6. Raman spectra of fluorinated CNF before and after Grinding

Table S1. Electrochemical data of F-Gr obtained at different reduction current densities

Discharge rate	Current density (mA/g)	Q_{exp} ± 10% (mAh/g)	$E_{1/2}$ (V)	Energy density (Wh/kg)	Power density (W/kg)	Faradic yield (%) ± 10%
0.01C	10	848	2.30	1950	23	108
0.05C	39	922	1.99	1835	78	117
0.2C	158	654	1.68	1099	265	83
1C	788	204	1.68	343	1324	26

Table S2. GR-Argon electrochemical data obtained at different reduction current densities

**Discharge rate**	**Current density (mA/g)**	**Q_{exp} ± 10% (mAh/g)**	**$E_{1/2}$ (V)**	**Energy density (Wh/kg)**	**Power density (W/kg)**	**Faradic yield (%) ± 10%**
0.01C	10	818	.34	1914	23	102
1C	801	493	1.90	937	1522	61

The electrochemical results for F-Grand F-Gr-Ar are in line with the physicochemical characterizations discussed above. The milling conditions used did not result in any significant modifications in terms of fluorination rate, content of sp^2 carbons, and C-F bond strength. After milling, the sample still possesses purely covalent C-F bonds, making the material insulating. However, an increase in the reduction potential is observed after milling under Argon. As the graphite particles, initially sub-fluorinated with an F/C equal to 0.8, have been broken and thus reduced in size by milling (SEM images Figure SI1), it is

conceivable that the non-fluorinated parts of the graphite are more exposed and thus allow better electronic conduction during the electrochemical process. However, the milled samples are not adapted to withstand high discharge regimes, limited to 1C, as is known from fluorinated carbons of the (CF)n type. Power and energy densities reach only 1521 W/Kg and 937 Wh/kg respectively.

References

S1. M. Colin, S. Chen, H. Farhat, K. Guérin, et M. Dubois, « Transparent wafer-scale self-standing fluorinated graphene films », Carbon, vol. 202, p. 137-149, janv. 2023, doi: 10.1016/j.carbon.2022.10.082.
